# Comparing BMD‐derived genotoxic potency estimations across variants of the transgenic rodent gene mutation assay

**DOI:** 10.1002/em.22137

**Published:** 2017-09-25

**Authors:** John W. Wills, George E. Johnson, Hannah L. Battaion, Wout Slob, Paul A. White, B. Gollapudi

**Affiliations:** ^1^ Environmental Health Science and Research Bureau, Health Canada Ottawa Ontario Canada; ^2^ Department of Biology University of Ottawa Ottawa Ontario Canada; ^3^ Institute of Life Science, Swansea University Medical School Swansea United Kingdom; ^4^ Department of Chemistry and Biomolecular Sciences University of Ottawa Ottawa Ontario Canada; ^5^ National Institute for Public Health and the Environment (RIVM) Bilthoven The Netherlands

**Keywords:** genetic toxicology, benchmark dose, transgenic rodent gene mutation assay, dose response analysis, human health risk assessment

## Abstract

There is growing interest in quantitative analysis of *in vivo* genetic toxicity dose‐response data, and use of point‐of‐departure (PoD) metrics such as the benchmark dose (BMD) for human health risk assessment (HHRA). Currently, multiple transgenic rodent (TGR) assay variants, employing different rodent strains and reporter transgenes, are used for the assessment of chemically‐induced genotoxic effects *in vivo*. However, regulatory issues arise when different PoD values (e.g., lower BMD confidence intervals or BMDLs) are obtained for the *same* compound across different TGR assay variants. This study therefore employed the BMD approach to examine the ability of different TGR variants to yield comparable genotoxic potency estimates. Review of over 2000 dose‐response datasets identified suitably‐matched dose‐response data for three compounds (ethyl methanesulfonate or EMS, *N*‐ethyl‐*N*‐nitrosourea or ENU, and dimethylnitrosamine or DMN) across four commonly‐used murine TGR variants (Muta™Mouse *lacZ*, Muta™Mouse *cII*, *gpt* delta and BigBlue® *lacI*). Dose‐response analyses provided no conclusive evidence that TGR variant choice significantly influences the derived genotoxic potency estimate. This conclusion was reliant upon taking into account the importance of comparing BMD confidence intervals as opposed to directly comparing PoD values (e.g., comparing BMDLs). Comparisons with earlier works suggested that with respect to potency determination, tissue choice is potentially more important than choice of TGR assay variant. Scoring multiple tissues selected on the basis of supporting toxicokinetic information is therefore recommended. Finally, we used typical within‐group variances to estimate preliminary endpoint‐specific benchmark response (BMR) values across several TGR variants/tissues. We discuss why such values are required for routine use of genetic toxicity PoDs for HHRA. Environ. Mol. Mutagen. 58:632–643, 2017. © 2017 Her Majesty the Queen in Right of Canada. Environmental and Molecular Mutagenesis Published by Wiley Periodicals, Inc.

## INTRODUCTION

Genetic toxicity testing is an essential component of safety assessments for new and existing substances (e.g., food additives, therapeutic products, pesticides, industrial chemicals). Its goal is to identify genotoxic substances and/or assess genotoxic potency, thus permitting regulatory decisions that minimize the risk of adverse human health effects (e.g., cancer and heritable genetic disorders) mediated by genetic damage. Traditionally, genetic toxicity tests have been interpreted *qualitatively*, with yes‐or‐no calls used to merely identify agents that have the ability to cause genetic damage (e.g., mutations and/or chromosomal aberrations). Increasingly however, there is growing interest in moving beyond binary categorizations that fail to acknowledge and appreciate variations in the genotoxic potency of tested agents (i.e., variations in the magnitude of the effect associated with a given dose). The alternative *quantitative* methods employ statistical analyses of genotoxicity dose‐response data to determine a point‐of‐departure (PoD) that provides quantitative information regarding genotoxic potency. Resultant PoDs, such as the lower confidence interval of the benchmark dose (i.e., the BMDL), can be used as a basis for quantitative risk assessments, including the derivation of human exposure limits and/or margins of exposure (MOEs). Thus, employment of quantitative methods, which acknowledge the relevance of genetic toxicity as a *bona fide* regulatory endpoint, permit the determination of values that can be used for human health risk assessment (HHRA) and regulatory decision‐making. Indeed, recent evaluations by several expert working groups have acknowledged the regulatory utility of quantitative dose‐response analyses of genetic toxicity data [Johnson et al., 2015; MacGregor et al., 2015a, 2015b; White and Johnson, 2016].

The transgenic rodent (TGR) gene mutation assays are well‐established *in vivo* assays for the assessment of chemically‐induced genetic toxicity. Critically, they provide reliable measurements of dose‐related inductions of gene mutations in practically any tissue. Briefly, TGR assays employ transgenic rodents (e.g., rats or mice) harboring multiple copies of stable, chromosomally integrated plasmids or bacteriophage shuttle vectors to determine a test article's ability to induce mutations at a transgenic target locus (e.g., *lacI*, *lacZ*, *cII*, and *gpt*). Following exposure to the test article, transgene mutations in selected tissues are detected by retrieving the vector and determining the phenotype of the reporter gene in a bacterial host [Douglas, 2010; Lambert et al., 2005].

In 2011, the Organisation for Economic Co‐operation and Development (OECD) published a test guideline (i.e., TG 488) for estimating the induction of *in vivo* somatic and germ cell gene mutations using TGR assays, thus contributing to the harmonization of TGR protocols employed to assess chemically‐induced, *in vivo* genetic damage [OECD, 2011]. The detailed review paper that preceded TG 488 noted that multiple variants of the TGR assay, which employ different rodent species and/or reporter transgenes, are available for regulatory evaluations of test articles [Lambert et al., 2005]. More specifically, when TG 488 was released there was considered sufficient information to demonstrate the utility of the following TGR assay variants: Muta™Mouse (*lacZ* and *cII* transgenes), Big Blue^®^ mouse and rat (*lacI* and *cII* transgene), plasmid mouse (*lacZ*), and *gpt* delta mouse and rat (*gpt*) [Douglas, 2010]. The results generated using these TGR assays constitute a useful follow‐up of *in vitro* positives; moreover, TGR results can augment regulatory decision‐making when cancer bioassay results are absent, marginal or inconclusive [Douglas, 2010; Lambert et al., 2005; OECD, 2011; Soeteman‐Hernández et al., 2016].

The value of the TGR assay for the evaluation of human health risks resultant from exposure to genotoxic substances was recently demonstrated in a landmark case involving human exposure to ethyl methanesulfonate (EMS), a potent mutagenic carcinogen. More specifically, between March and June 2007 a batch of Viracept^®^ (i.e., nelfinavir mesylate), an antiretroviral protease inhibitor, was accidently contaminated with EMS, resulting in a global recall of the drug [Pozniak et al., 2009]. Since existing data was considered inadequate for effective patient risk management, a comprehensive Muta™Mouse study of EMS was carried out. The analyses scored micronucleus frequency in bone marrow and *lacZ* mutations in selected tissues (e.g., small intestine, liver, bone marrow) following 28‐day repeat dose oral exposure. The results were used to demonstrate that the minimal dose required to elicit a significant increase in the Muta™Mouse response (i.e., 25 mg/kg/day) is approximately ∼450‐fold greater than the maximum amount ingested by patients receiving the product (i.e., 0.055 mg/kg/day). These analyses were used to conclude that the risk of adverse health effects in individual patients receiving the contaminated product were negligible [Muller and Gocke, 2009; Muller, et al. 2009; Pozniak et al., 2009)].

**Figure 1:**
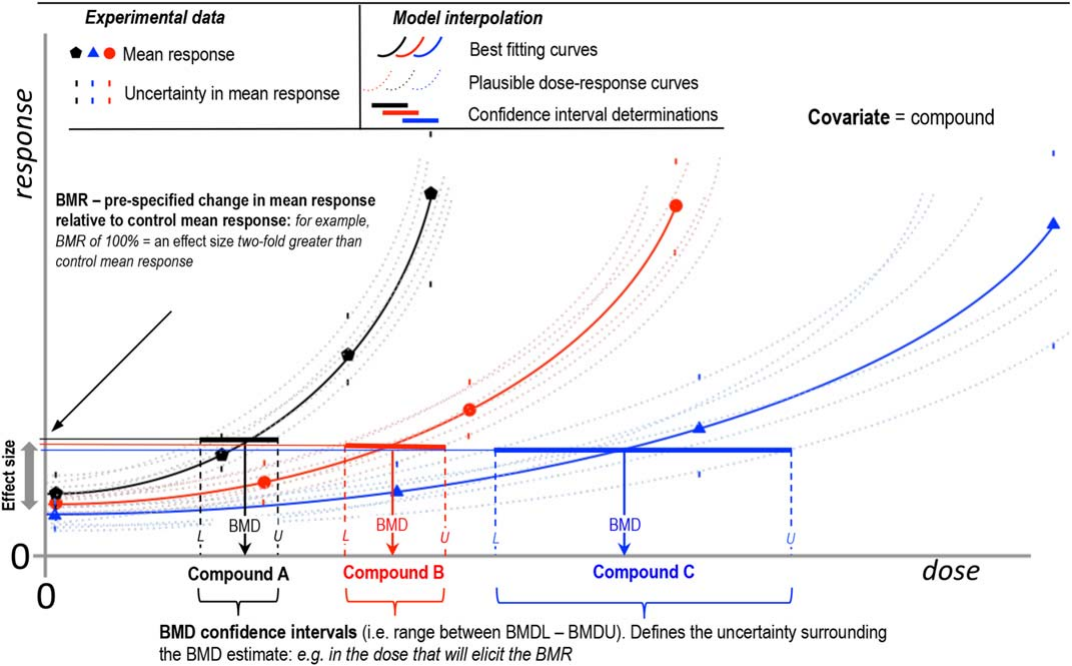
Schematic overview of the BMD combined covariate approach. The benchmark dose approach (BMD) provides an estimate of the dose that will elicit a small, pre‐specified effect‐size called the benchmark response (BMR). The best‐fitting BMD model (solid curve) will result in the best estimate of the BMD. Importantly however, the uncertainty in the dose‐response data needs to be accounted for through calculation of a BMD confidence interval. The process can be conceptually visualized by imagining that, through variation of the model parameters, other curves, and BMDs that plausibly describe the data (e.g., schematically represented by the dashed curves) may be established. Together these values comprise the BMD confidence interval (solid line). In turn, this process provides estimations of the BMDL (L) and BMDU (U), the lower and upper 90% confidence bounds of the BMD estimate, respectively. Therefore, the ratio of the BMDU to BMDL represents the *precision* to which the true BMD can be estimated based on the available dose‐response data. A newer method termed the combined, BMD‐covariate approach allows BMDs for multiple dose‐response curves to be defined in one combined analysis employing a covariate such as compound, exposure regime, time, sex, or species to identify the constituent dose‐response relationships. The major advantage of this approach is that any model parameters that are determined to be similar across covariate subgroupings may be held constant during an analysis; thus their estimation is based on *all* the dose‐response curves included in the combined analysis. Concomitantly, the precision of the BMD estimate is improved (i.e., reduced BMDU‐to‐BMDL ratio) for any individual dataset under consideration.

The Viracept^®^ contamination incident stimulated the formation of several international working‐groups that evaluated different statistical approaches for quantitative analysis of genetic toxicity dose‐response data [Gollapudi et al., 2013; Johnson et al., 2014, 2015; MacGregor et al., 2015a, 2015b]. These works led to general agreement that the benchmark dose (BMD) approach, which is increasingly used for the evaluation of other toxicity dose‐response data, also provides the most appropriate method for the quantitative interpretation of genetic toxicity dose‐response data [Gollapudi et al., 2013; Johnson et al., 2014; MacGregor et al., 2015a, 2015b]. The BMD approach involves statistical analysis of the dose‐response relationships to define the dose (i.e., the BMD) required to elicit a pre‐specified small increase in response (i.e., the BMR or Benchmark Response) [Crump, 1984; EFSA, 2009, 2017; Slob, 2002]. Figure 1 schematically illustrates the BMD approach, and how the BMD can be used to quantitatively define genotoxic potency. As data are always limited (i.e., limited number of doses and replicates) the true BMD can never be defined *exactly*, and hence reporting a BMD confidence interval that can be expected to include the true BMD with a defined level of confidence is preferable over reporting of a single point estimate of the BMD. The lower and upper bounds of the BMD confidence interval are called BMDL and BMDU, respectively. The ratio of BMDU‐to‐BMDL thus reflects the uncertainty (i.e., imprecision) in the BMD estimate in accordance with the quality of the underlying data [Wills et al., 2016a].

More recent advances of the BMD methodology permit simultaneous analyses of multiple dose‐response datasets for a common endpoint and study design (Fig. 1) in one *combined* analysis. This approach, which is known as the combined BMD covariate method, uses inclusion of a covariate (e.g., compound, sex, species, etc.) to identify constituent dose‐response sub‐groupings [Slob, 2002; Slob and Setzer, 2014]. Critically, it has been shown that this combined approach can improve the *precision* of each individual BMD estimate (i.e., bring about a reduction in BMDU‐to‐BMDL ratios) as all dose‐response datasets included in a combined analysis contribute information to any individual BMD estimate under consideration [Slob and Setzer, 2014; Wills et al., 2016a,b].

Following on from the aforementioned Muta™Mouse (*lacZ*) study of EMS (i.e., analyses related to the Viracept® contamination incident), a 2014 study repeated the work using a matching study design, but, instead employed the *gpt* delta mouse (*gpt* transgene) TGR assay [Cao et al., 2014]. The specific aim of this work was to utilize quantitative methods to compare genotoxic potency estimates for two TGR assay variants with well characterized differences in spontaneous mutant frequency (i.e., background). The authors noted that the tissue‐specific BMDL_10_ values associated with the *gpt* delta mouse data were lower than those calculated from Muta™Mouse data. Subsequently, these divergent results triggered an international response from multiple working groups regarding the regulatory issues associated with differences in BMDL values obtained across different TGR assay variants.

In response to these concerns about cross‐study differences in the transgenic rodent BMDL values for EMS, the Quantitative Analysis Workgroup of the International Life Sciences Institute (ILSI) Health and Environmental Sciences Institute (HESI) Genetic Toxicology Technical Committee (GTTC) prioritized quantitative examination of published TGR dose‐response data. More specifically, quantitative analyses assessing the ability of different TGR assay variants to yield comparable estimates of genotoxic potency were established as a priority. This work required an initial meta‐analysis of all available TGR data to identify compounds, including EMS, that have been tested using two or more TGR assay variants. To ensure robust comparisons, datasets were screened to identify those that are also suitably matched with respect to tissue, route of administration, application schedule, and sampling time. Herein, we thus use the BMD approach to evaluate the quantitative agreement in genotoxic potency estimates resulting from employment of different TGR assay variants. We also assess and discuss the importance of appropriately defining benchmark response values (BMRs), also known as critical effect sizes (CES).

## MATERIALS AND METHODS

### Data Collection

TGR assay dose‐response data were obtained from the latest version of the Transgenic Rodent Assay Information Database (TRAID). TRAID is a database containing dose‐response data for all publicly available TGR studies published as of August 31, 2016. The current version, which was updated by screening PubMed and Scopus using the search string ((“MutaMouse” OR “Big Blue” OR “lacZ” OR “lacI” OR “*gpt*” OR “cII”) AND (mutation OR mutant OR mutagen OR mutagenesis)) AND (mouse OR rat OR rodent OR mice OR rats OR rodents), is an updated version of the original database compiled by Lambert et al. (2005). Briefly, the database contains 9716 records representing 2127 dose‐response datasets from 406 studies on 307 test articles, including dose‐response data for five transgenes (e.g., *lacZ*, *lacI*, *cII*, *gpt*, and red/gam), 31 tissues, and 25 administration routes. The complete dataset was screened using SAS v9.4 to identify suitably matched dose‐response data for compounds that have been examined using more than one TGR assay. For the purposes of this study, compound‐specific dose‐response data were only retained if data were available in two or more TGR assay variants, if the studies used three or more dose‐groups (i.e., suitable for BMD modeling), and if the datasets could be matched for tissue, route of administration, exposure duration, and sampling time. This stringent screening criteria revealed only five compounds that have been tested in two or more TGR assays using analogous study design (i.e., EMS, *N*‐ethyl‐*N*‐nitrosourea or ENU, chlorambucil, dimethylnitrosamine or DMN, and diethylnitrosamine or DEN). Of these, 14 dose‐response datasets across three of the compounds (i.e., EMS, ENU, and DMN) utilized a sufficient number of dose groups (3+) to be suitable for BMD analysis. The full extent of dataset matching in terms of study covariates for each presented analysis is presented in Supporting Information Table SI, alongside the data‐source citations.

### BMD Analysis

BMD analyses (i.e., statistical analysis of the dose‐response data) were conducted using PROAST software version 63.6 in the R computing environment (Dutch National Institute for Public Health and the Environment (RIVM)). PROAST v38.9 is available for free download at http://www.proast.nl. More recent versions (e.g., 63.6) can be obtained from one of the authors (i.e., Wout Slob). Datasets were analyzed using both the exponential and the Hill model families as recommended by the European Food Safety Authority (EFSA) for the assessment of continuous data [EFSA, 2009, 2017]. Where the combined BMD‐covariate approach was used, the factor discriminating the dose‐response subgroupings was included as covariate (e.g., study or transgene). For the combined BMD‐covariate approach, the model parameters that require estimation for each subgroup, alongside those that can be considered constants across subgroups, are determined. Combined analyses typically assumed that model ‘shape’ parameters ‘*c*’ and ‘*d*’ (i.e., maximum response and log‐steepness after axis scaling) were constant across subgroups, whilst the parameters ‘*a*’, ‘*b*’, and ‘*var*’ (background response, potency, and within‐group variation, respectively) were tested for subgrouping dependency. BMD analyses and model fits for each subgrouping are presented in Supporting Information Figures S1‐S4, with the model fits used to visually assess the validity of the conserved shape assumption. This approach was preferred to statistical testing as statistical tests on the BMD model shape parameters (i.e., c, d) have been shown to be extremely sensitive to the non‐random errors that are ubiquitously present in experimental dose‐response data since it is not practically feasible to randomize all experimental conditions and concomitant treatments. Critically, due to the statistical power arising in a combined dataset, even small non‐random errors in the data can result in rejection of shape parameter consistency. In reality however, such small differences in shape parameters can only, at most, have a very minor effect on the coverage of the calculated confidence intervals [Slob and Setzer, 2014].

The BMDL and BMDU values represent the two‐sided, lower and upper 90% BMD confidence intervals, respectively, thus the BMDU‐to‐BMDL ratio defines the BMD estimate precision. Confidence interval plots were employed to visually compare differences in potency, in order to take estimation uncertainty into account [Bemis et al,. 2016; Wills et al., 2016a, 2016b]. Dose‐response relationships across subgroupings (e.g., different TGR assay variants) can only be termed significantly different where confidence intervals do not overlap. Where employed, endpoint‐specific BMR values were calculated in the PROAST software as the control‐group mean plus one typical standard deviation. Briefly, this typical value of the standard deviation, which was calculated on the log‐scale, was defined as the average within‐group standard deviation calculated across all dose‐groups and studies included in a combined BMD analysis. The full rationale for this choice, and the importance of its calculation on the log‐scale, is discussed at length in Slob (2017) and its accompanying annexes. To facilitate comparisons to fixed percentage effect sizes (e.g., BMR = 10%), resultant endpoint‐specific BMRs were expressed as percentage increases relative to the control‐group mean. The calculated BMD values underlying the confidence interval plots show in each analysis are provided in Supporting Information Table SII.

## RESULTS AND DISCUSSION

### A Survey of TGR Studies to Identify Matched Dose‐Response Datasets

In order to compare genotoxic potency estimates derived from dose‐response data generated using different variants of the TGR assay (i.e., different strains and/or transgenes), a meta‐analysis filtration of the TRAID database was performed (see Methods) to identify suitably‐matched datasets (i.e., in terms of compound, species, tissue, administration route, exposure duration, sampling time) that are thus well‐suited for potency estimate comparisons. Scrutiny of over 9700 records containing over 2000 dose‐response datasets identified only 14 datasets that fulfilled the screening criteria (detailed in Supporting Information Table SI). The comparison of these matched studies form the basis of the results presented herein.

In consideration of the findings of the meta‐analysis, the relatively low number of matching datasets is indicative of the variability of study designs historically employed within and between different TGR assay variants. Thus, these findings alone suggest the importance of global harmonization initiatives (e.g. OECD TG 488) in facilitating the maximum utility of TGR dose‐response data. Cross‐study comparisons are simplified when results are generated using analogous methods and study design as this dramatically reduces the number of potentially influential covariates. In turn, consistent dose‐response data are also better‐suited to support human health risk assessments, whereby findings are often compared across studies during the process of weight‐of‐evidence based regulatory decision‐making [Dearfield et al., 2017; MacGregor et al., 2015a].

### Use of the BMD Approach to Compare Potencies and Assess Inter‐Study Reproducibility

In previous publications, we showed that comparisons of BMD confidence intervals (see Fig. 1), as opposed to direct comparison of single metric (i.e., BMD, BMDL or BMDU), constitutes a robust way to quantitatively examine differences in potency across dose‐response relationships [Bemis et al., 2016; Soeteman‐Hernández et al., 2016; Wills et al., 2016a, 2016b]. Considering BMD confidence intervals for the purposes of comparisons between studies is critical, since they reflect the *uncertainty* in the potency estimate as a result of the uncertainties in the underlying dose‐response data (e.g., due to random sampling errors between replicates). Consequently, one can only conclude that dose‐response relationships and potency estimates (e.g., BMDs) arising from, for example, assessments of the same compound across different variants of the TGR assay, are significantly different when their BMD confidence intervals do not overlap [Wills et al., 2016a,b]. In contrast, overlapping confidence intervals signify that any potency differences cannot be resolved on the basis of the available data (i.e., the true BMDs could be the same, or could differ). Critically, when the range delineated by all the confidence intervals examined is small enough to be considered as biologically or practically insignificant, it may be concluded that the potencies are sufficiently similar to consider the associated studies reproducible [Johnson et al., 2016; Wills et al., 2016a,b].

### EMS: Comparisons of *gpt*‐Delta Mouse (*gpt*) and Muta™Mouse (*lacZ*) Dose‐Response Data for Bone Marrow, Small Intestine and Liver

In their analysis of EMS dose‐response data across two TGR variants (Muta™Mouse (*lacZ*) and *gpt* delta mouse), Cao et al., (2014) noted lower BMDL_10_ values associated with the *gpt* delta mouse data (see introduction). Critically however, this finding cannot be interpreted as evidence of poor potency estimate comparability between the two TGR assay variants, since, as noted, potency comparisons should not be based on any single BMD or BMDL value, but rather on comparisons of complete BMD confidence intervals. In their combined BMD analysis, Cao et al., (2014) also used ‘tissue’ as a dose‐response covariate: this choice of covariate is potentially problematic since any correlation between tissues harvested from the same animals could give rise to overly optimistic (i.e., narrow) BMD confidence intervals. Thus, in the analyses presented below, we chose to carry out pairwise combined BMD analyses tissue‐by‐tissue using ‘TGR’ as covariate.

**Figure 2:**
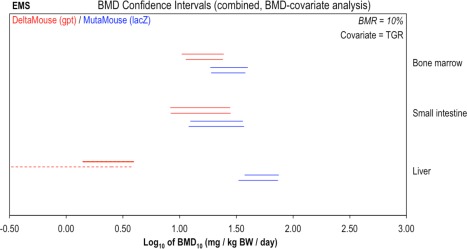
Genotoxic potency of ethyl methanesulphonate (EMS) determined using the *gpt* delta Mouse (red) and Muta™Mouse (blue) transgenic rodent assays. BMD analyses, with TGR as covariate, were conducted to compare potency values (i.e., BMDs) determined using two different TGR assay variants. Two‐sided 90% confidence intervals of the BMD_10_ (i.e., BMR = 10%) were calculated from mutant frequency (MF) dose‐response data (i.e., *gpt* or *lacZ* MF) for bone marrow, small intestine, and liver tissues using two different BMD models: the exponential (upper interval per pair) or the Hill (lower interval per pair). Combined analyses were performed, two datasets at a time by tissue, using TGR as covariate. The dashed liver interval for the Hill model indicates an unbounded lower confidence limit (i.e., BMDL = 0). The underlying dose‐response data and fitted BMD models are presented in Supporting Information Figure S1.

Applying this approach (i.e., confidence interval comparison and TGR as covariate), Figure 2 presents a reanalysis of the EMS dose‐response data for the *gpt* delta mouse [Cao et al., 2014] and Muta™Mouse [Gocke et al., 2009] TGR systems. Consideration of the bone marrow and small intestine results shows that whereas the *gpt* delta mouse BMDL_10_ values are consistently lower than the matched Muta™Mouse values, the confidence intervals show considerable overlap. This means that the BMDs could, in fact, be identical and thus the available data do not allow us to conclude that the assay results are non‐reproducible. In turn, the BMD confidence intervals also give an indication of how large the differences between the assays might be. In this example, the range spanned by the confidence intervals for bone marrow and small intestine indicate that, at most, the genotoxic potency estimates may differ by ∼0.6 log units. Thus, on the basis of the available data, it can only be concluded that the difference in potency estimates arising across these two assays may be anywhere between zero and 0.6 log units (i.e., 0 to ∼4‐fold).

Interestingly, comparison of the liver data shows an infinite lower bound of the BMD (represented by the dashed interval) for the *gpt* delta mouse data when the BMD_10_ was calculated using the Hill model, whereas the BMDL was calculable using the exponential model. An infinite lower bound indicates a dose‐response relationship whereby the data only allow us to conclude that *any* dose greater than zero might elicit the specified BMR (i.e., 10% for this analysis). Thus, such a finding typically reflects uncertainty in the dose‐response data within the response region interpolated at the effect size (i.e. BMR). Closer scrutiny of the liver datasets (Supporting Information Figure S1) illustrates that the two TGR variants show opposite dose‐response patterns. More specifically, for the *gpt* delta mouse, the mean response in the lowest dose‐group is higher than control, yet the response does not subsequently increase further with increasing dose. In contrast, for the Muta™Mouse dataset, only the mean response in the highest dose‐group indicates a response that is marginally increased relative to background. Thus, it seems likely that for the *gpt* delta mouse liver dataset, the control‐group response is an outlier and that no true dose‐response is established across the tested dose‐range. Similarly, for the Muta™Mouse liver data set, the highest dose‐group might represent an outlier. Thus, without further (i.e., higher) dose‐groups to clearly establish the dose‐response relationships in this tissue, these datasets are not suitable for dose‐response analysis. This uncertainty is reflected in the aforementioned unbounded BMD confidence interval for the *gpt* delta mouse data, whereas the confidence interval established from the Muta™Mouse liver data shows that, even if the apparent response in the highest dose group *is* a true dose‐related effect, liver is less sensitive than the other two tissues. In a wider context, the difference between the exponential and Hill model confidence intervals arising from the *gpt* delta mouse liver data exemplifies the importance of carrying out BMD analyses using both model families (i.e., exponential and Hill) to ensure the uncertainty in BMD estimates is thoroughly characterized. This is particularly important when a robust estimate of genotoxic potency is essential for (e.g. for HHRA purposes), as has been advocated previously [EFSA, 2016; Johnson et al., 2014; MacGregor et al., 2015b; Slob and Setzer, 2014].

When carrying out BMD analyses on large combined datasets, the choice of the BMR percentage has been shown to be fairly unimportant for the purposes of objectively comparing genotoxic potencies [Bemis et al., 2016]. However, each combined analysis conducted herein only involved a small number of datasets, thus the precision of the BMD estimate decreases with choice of smaller and thus more difficult‐to‐estimate effect sizes (i.e., smaller BMRs). Indeed, in the above EMS analysis, the BMR of 10% was more or less arbitrarily chosen on the basis of no more than the fact that it is often used in the analysis of toxicological dose‐response data. On the other hand, when the purpose of the analysis is to derive BMD values for HHRA purposes, it becomes necessary to define a *defensible, meaningful* small BMR size for that can be utilized as a basis for the robust determination of HHRA metrics (e.g., permitted daily exposure or PDE, MOE, tolerable daily intake or TDI, etc.). To this end, Slob (2017) recently outlined a statistical framework that can be used to determine meaningful BMRs for toxicological endpoints. The theory predicts a general relationship between the maximum response (i.e., maximum fold change in response relative to control) and within‐group variation (i.e., variability in response measurements obtained between animals in a dose‐group) for any toxicological endpoint. Demonstrating this relationship using dose‐response data for ∼27 different endpoints, the work thus provides a basis for setting BMRs appropriately in context of each endpoint's response maximum. Thus, the theory supports the underlying rationale of the BMR 1‐standard deviation or BMD_1SD_ approach (i.e., where the control group mean plus one standard deviation is employed to define the BMR; thus accounting for differences in the ‘natural variability' across different endpoints) [Slob, 2017]. However, due to the ubiquitous errors present in experimental measurements (e.g., due to limited replications, differences between experimental animals etc.), instead of using the observed within‐group standard deviation of a specific study as the effect size, the theory demonstrates that it is better to use the *typical* value of the standard deviation. In other words, averaged across all dose‐groups and across a large number of studies per endpoint. To this end, ongoing efforts are establishing large datasets that will permit robust estimation of the within‐group variation for selected endpoints (e.g., *in vivo* micronucleus, *pigA*, and tissue‐specific transgenic rodent assay tests). Since this work has yet to be completed, in applying the Slob (2017) approach herein, we are limited to using a rough estimate of the typical within‐group variance based on the few datasets at hand to define meaningful endpoint‐specific BMRs. The values employed in the below analyses should not yet, therefore, be regarded as robust endpoint‐specific BMR values that can be used for regulatory purposes, but rather as a preliminary estimate with the purpose of employing a more reasoned choice for the BMR than just selecting an arbitrary value such as 10%.

**Figure 3:**
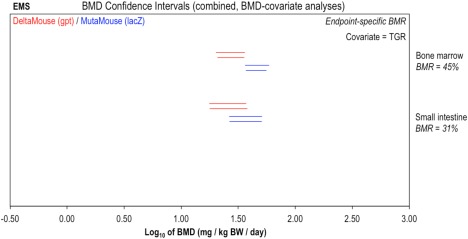
Genotoxic potency of ethyl methanesulphonate (EMS) determined using the *gpt* delta mouse (red) and Muta™Mouse (blue) transgenic rodent assays: endpoint‐specific BMRs. BMD analysis, with endpoint‐specific BMR values, was conducted to compare BMDs determined across two TGR assay variants. Two‐sided 90% confidence intervals of the BMD, based on BMRs indicated beneath each tissue, were calculated from mutant frequency (MF) dose‐response data (i.e., *gpt* or *lacZ* transgene) for bone marrow, small intestine, and liver tissues using two different BMD models: the exponential (upper interval per pair) or the Hill (lower interval per pair). Combined analyses were performed, two datasets at a time by tissue, using TGR as covariate. The underlying dose‐response data and fitted BMD models are presented in Supporting Information Figure S2.

Applying the aforementioned Slob (2017) approach to the EMS *gpt* delta and MutaMouse^TM^ (*lacZ*) datasets yielded endpoint‐specific BMRs of 45% and 31% for bone marrow and small intestine, respectively (Fig. 3). In comparison to the analyses using a BMR of 10% (Fig. 2), these increases in effect‐size (BMR) are seen to improve the precision in the BMD estimates (i.e. reduce the BMDU‐to‐BMDL ratios). This is as expected, since the increased BMR results in interpolation of the data *further up* the dose‐response curve; closer to the range of experimental observation where concomitantly, the dose‐response relationship is less uncertain (the reader is referred to the introduction / Figure 1 for a full visual explanation of the BMD approach and derivation of the BMD confidence interval). For small intestine, genotoxic potency estimates from the two TGR variants span only ∼0.5 log units (i.e., factor of ∼3), and show considerable overlap, suggesting good reproducibility. In contrast, the bone marrow confidence intervals are now sufficiently well‐resolved to be distinct from one another, indicating a slightly lower genotoxic potency estimate from the *gpt* delta mouse data in comparison with Muta™Mouse. However, before concluding that these non‐overlapping confidence intervals are indicative of poor TGR variant reproducibility, it is important to note that the ranges encompassed by the BMD confidence intervals are only ∼0.5 log units at most, so, the difference in BMDs is somewhere between ∼0 and 0.5 log units (i.e., again, at most, a factor of 3). BMD model fits to the data are presented in Supporting Information Figure S2.

### ENU: Comparisons of Muta™Mouse (*lacZ* or *cII*) and *gpt* Delta Mouse Small Intestine Dose‐Response Data

**Figure 4:**
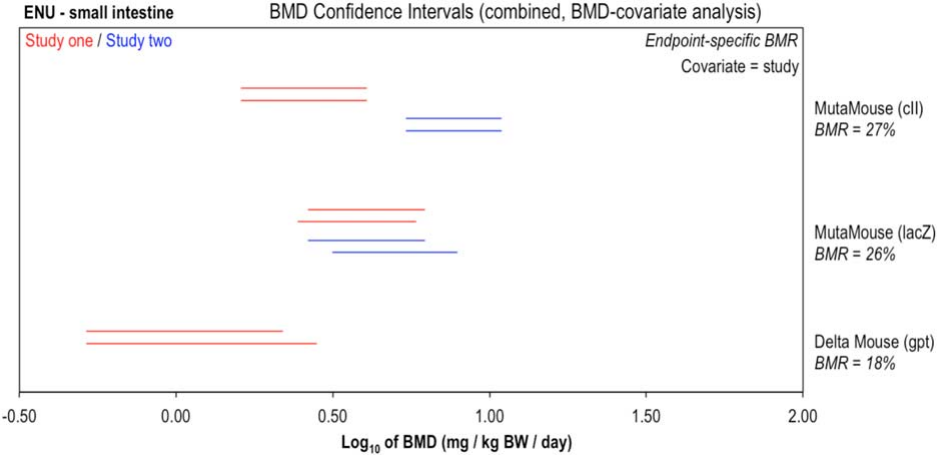
Genotoxic potency analysis of *N*‐ethyl‐*N*‐nitrosourea (ENU) determined using the Muta™Mouse (*cII* or *lacZ* transgenes) or *gpt* delta Mouse (*gpt* transgene) transgenic rodent assays. BMD analysis, with endpoint‐specific BMR values, was conducted to compare BMDs determined using two TGR assay variants. Two‐sided 90% confidence intervals of the BMD, based on BMRs indicated beneath each tissue, were calculated from mutant frequency (MF) dose‐response data (i.e., *gpt* or *lacZ* transgene) for bone marrow, small intestine, and liver tissues using two different BMD models: the exponential (upper interval per pair) or the Hill (lower interval per pair). Combined analyses were performed, two datasets at a time by tissue, using study as covariate. The underlying dose‐response data and fitted BMD models are presented in Supporting Information Figure S3.

Moving to a different compound, ethyl nitrosourea (ENU), Figure 4 presents a comparison of the potency estimates derived from matched Muta™Mouse (*cII* and *lacZ* transgenes) and *gpt* delta mouse datasets. Here, matching data from two independent studies were further available for each of the Muta™Mouse assessments. BMD analyses were carried out by transgene using study as covariate, with endpoint‐specific BMR values estimated from the typical within‐group variances across the available studies for each transgene (i.e., as outlined above for EMS). When considering the resultant confidence intervals, it is interesting that the two Muta™Mouse (*lacZ*) studies show highly similar potency estimates, whilst the data arising from the two Muta™Mouse *cII* studies generated significantly different (i.e., non‐overlapping) confidence intervals. This shows that even when the same TGR assay variant is employed, differences in potency estimate can arise, presumably due to a variety of different experimental factors (e.g., litter, diets, housing, animal handling, etc.). In this specific instance, consideration of the data (Supporting Information Figure S3) shows that the major difference between the two *cII* datasets is related to the control‐group values, with the mean control group (i.e., background) responses differing by almost an order of magnitude. Interestingly, above the control, the mean responses for the groups of experimentally dosed animals are highly similar across the two studies. This suggests that the control‐group in one of the two studies likely constitutes an outlier attributable to an unknown experimental factor. With respect to the design of these studies, both were conducted using three dose‐groups plus control with six animals per dose‐group. The establishment of different potency estimates as a result of differing control‐group values therefore suggests that a more effective study design could have utilized a greater number of dose groups and fewer animals per group. If this had been the case, the influence of one outlying group on the calculated BMD confidence interval may have been reduced [Slob, 2014a; Slob, 2014b]. Regardless, consideration of the BMD confidence intervals also shows that the uncertainty in the genotoxic potency estimates caused by these different control‐group responses (i.e., lowest BMDL to highest BMDU across the two studies) is at worst ∼0.8 log units (factor of ∼6), and may in fact be considerably less. Applying the same logic to the complete set of confidence intervals, including those from the *gpt* delta mouse study, it can be seen that the genotoxic potency estimates arising from the three TGR variants *could* differ by up to ∼1 order of magnitude; however, it might also be that they differ by only ∼0.3 log units (i.e., a factor of ∼3: BMDU of *gpt* delta mouse to BMDL Muta™Mouse *cII* study two).

### DMN: Comparisons of BigBlue^®^ Mouse (*lacI*) and Muta™Mouse (*lacZ*) Liver Dose‐Response Data

**Figure 5:**
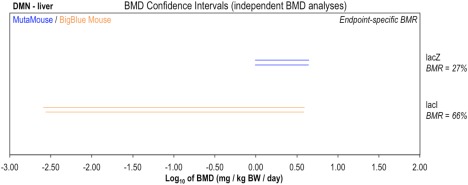
Genotoxic potency analysis of dimethylnitrosamine (DMN) determined using the Muta™Mouse (blue) or BigBlue^®^ Mouse (orange) transgenic rodent assays. BMD analysis, with endpoint‐specific BMR values, was conducted to compare BMDs determined using two TGR assay variants. Two‐sided 90% confidence intervals of the BMD, based on BMRs indicated beneath each tissue, were calculated from mutant frequency (MF) dose‐response data (i.e., *lacZ* or *lacI* transgene) in liver tissue using two different BMD models: the exponential (upper interval per pair) or the Hill (lower interval per pair). Datasets were analyzed individually. The underlying dose‐response data and fitted BMD models are presented in Supporting Information Figure S4.

The final matched datasets permit comparison of Muta™Mouse *lacZ* and BigBlue^®^ mouse *lacI* DMN dose‐response data for liver following exposures to dimethylnitrosamine (DMN) (Fig. 5). Whereas both study designs were two‐dose plus control with four animals per dose‐group the confidence intervals show much greater BMD estimate precision from the Muta™Mouse data in comparison to that from BigBlue^®^. The BMD analysis (Supporting Information Figure S4) indicates that this is related to the fact that the within‐group variation (*var* parameter) in the BigBlue^®^ data was ∼4‐fold greater than that associated with the Muta™Mouse data. Importantly, this shows that questions related to the number of animals required to suitably define a metric such as the BMD are not easily addressed. The precision of the metric is heavily influenced by the actual scatter in the experimental observations (i.e., response measurements) across animals. Thus, in this particular instance, the utility of the *lacI* study to compare the two assays' potency estimates is limited. This is reflected in the confidence intervals, which reveal that, based upon the available data, the difference between the BMDs could be up to ∼2 orders of magnitude. However, due to confidence interval overlap, it is also possible that the BMDs are in fact highly similar.

## CONCLUSIONS AND RECOMMENDATIONS

Despite considering the entire TRAID database, which contains over 2000 dose‐response datasets, the meta‐analysis only identified 14 suitably‐matched datasets that were thus ideally suited for purposes of comparing BMD‐derived potency estimates. The lack of suitably matched data inevitably places limitations on what can be concluded with regard to the question of potency estimate reproducibility across different TGR assay variants. With this in mind, none of the analyses presented herein across four commonly used murine TGR variants (i.e. Muta™Mouse *lacZ*, Muta™Mouse *cII*, *gpt* delta mouse, and BigBlue^®^ mouse *lacI*) revealed significantly different (i.e. non‐overlapping) BMD confidence intervals when assessing matched datasets for the same compound. This finding was reliant however upon taking into account the importance of comparing genotoxic potency estimates in context of dose‐response relationship *uncertainty*. More specifically, similar to our earlier works [e.g., Bemis et al. 2016; Wills et al. 2016a, 2016b], we demonstrate that comparing BMD confidence intervals, as opposed to the comparison of any single metric (e.g. BMD, BMDL or BMDU), is essential for robust comparisons of genotoxic potency estimates.

With these findings in mind, it is useful to note that our previous work comparing TGR‐derived BMDs across multiple tissues from Muta™Mouse specimens exposed to the prototypical mutagenic polycyclic aromatic hydrocarbon benzo[*a*]pyrene showed significant differences in genotoxic potency across four out of the five tissues analyzed (i.e. small intestine, bone marrow, glandular stomach, liver) [Wills et al., 2016b]. Moreover, the BMD confidence intervals established across these different tissues spanned a dose range of ∼1.5 orders of magnitude (i.e., a factor of ∼30). From a biological perspective, this seems hardly surprising given the differences in exposure dosimetry that will be established across tissues by the complex processes of adsorption, distribution and compound‐specific metabolism. Thus, for generation of comparable TGR‐derived potency estimates, questions regarding the tissue(s) that should be collected, analyzed, and subsequently used to derive a PoD, appear to be equally or potentially more important than questions related to the choice of TGR assay variant.

With respect to the selection of tissues that can provide data which are useful for regulatory evaluations of genotoxic substances, our results suggests that cryogenic storage of multiple tissues collected at necropsy is a recommended strategy to avoid the possible necessity of repeating a costly *in vivo* study. Regarding tissue choice, it is suggested that studying multiple tissues, ideally chosen based on known or hypothesized mode of action (MOA) information, inclusive of any knowledge of similar compounds' ADME (adsorption, distribution, metabolism, and excretion) kinetics, provides significantly greater safeguarding than just studying a single tissue. For example, despite metabolic competence, liver may not be ideal due to lower cell proliferation rates and elevated repair that may significantly reduce sensitivity to mutation [Wills et al., 2016b].

Finally, it is clear that whilst fixed‐percentage BMRs are effective for potency comparisons across covariates, the use of BMDs for HHRA requires specification of a meaningful, small BMR size. Consequently, this work argues that it is essential to tackle the determination of endpoint‐specific effect sizes (i.e. BMRs); especially now that a statistical framework for understanding the relative magnitude of effect sizes in dose‐response relationships is becoming established [Slob, 2017]. Use of this approach, in conjunction with the employment of advanced BMD methods such as the combined covariate approach, shows that the BMD method improves the utility of genetic toxicity dose‐response data by provision of robust, comparable estimates of genotoxic potency.

To summarize, this work offers several conclusions and recommendations. The first four relate to effective use of the BMD approach for analysis of genetic toxicity dose‐response data. The last two, which have been identified as priorities for the Health and Environmental Sciences Institute Genetic Toxicology Technical Committee (HESI‐GTTC), relate to more specific issues regarding the reproducibility of potency estimates across TGR assay variants, and the utility of genetic toxicity PoD estimates for risk assessment and regulatory decision‐making.

1. As noted in the aforementioned earlier works, the BMD approach provides robust estimates of genotoxic potency; moreover, the combined covariate approach can be used to investigate the influence of covariates such as tissue and assay variant on the derived potency estimates.
2. International guidelines that harmonize genotoxicity assay study designs increase the utility of dose‐response data, and thus the use of experimental animals beyond that of the originating study. This is achieved through a reduction in the number of influential covariates, which in turn facilitates subsequent cross‐study comparisons. However, it is suggested that more routine adoption of study designs employing fewer animals per dose group, alongside a greater number of dose‐groups, could further improve the quantitative utility of dose‐response data by reducing the influence of any single outlying group on the calculated BMD confidence interval.
3. Determining BMDs and associated confidence intervals, via both the exponential and the Hill model families, is a recommended best‐practice to ensure that the uncertainty in the estimated BMD is appropriately defined. Moreover, comparisons of potency across covariates should not be based on one single value such as the BMD, BMDL or BMDU. Rather, effective comparisons across covariates, such as assay variant or tissue, requires consideration of BMD confidence intervals.
4. The relative strengths and weaknesses of the BMR_%_ (i.e., percentage increase above background) and BMR_1SD_ (i.e., BMR = control‐group mean plus one control‐group standard deviation) approaches can be reconciled via determination of endpoint‐specific BMR_%_ values. These BMRs should be derived from the typical endpoint‐specific maximum response or the typical endpoint‐specific within‐group variation values i.e., based on a large number of studies for the same endpoint. Efforts to achieve this for a range of genetic toxicity endpoints are currently underway.
5. With respect to the reproducibility of genotoxic potency estimates, we found no evidence that the choice of TGR assay variant significantly influences compound‐specific BMD determination. Based on these and earlier results, it might be hypothesized that the choice of tissue for a given TGR assay variant is equally, or potentially more important, than the choice of TGR variant itself. Thus, cryogenic storage of numerous tissues is recommended, as is genotoxic potency analyses in multiple tissues selected in consideration of supporting information (e.g. MOA, ADME kinetics).
6. There is currently no consensus regarding the most pragmatic methodology to routinely employ *in vivo* genotoxicity potency estimates for regulatory decision‐making. Thus, it will be necessary to scrutinize the results of the current study, as well as our earlier studies and related studies by other authors, to determine the most appropriate approach. Indeed, some international advisory groups are currently considering issues regarding the use of BMD values for regulatory evaluation of genotoxic test articles such as pharmaceutical impurities and food contaminants [Benford, 2016; ICH, 2014, 2015].

## AUTHOR CONTRIBUTIONS

JWW, GEJ, and WS analyzed the data. PAW and HLB carried out the meta‐analysis of published TGR dose‐response data. JWW wrote the manuscript in collaboration with PAW, WS, and GEJ. All authors reviewed the manuscript and approved submission.

## Supporting information

Supporting Information Fig S1

Supporting Information Fig S2

Supporting Information Fig S3

Supporting Information Fig S4

Supporting Information Tab S1

Supporting Information Tab S2
