# Supplementary figures and images for: Comparing BMD‐derived genotoxic potency estimations across variants of the transgenic rodent gene mutation assay
Source: Environ Mol Mutagen. 2017 Sep 25;58(9):632–43. doi: 10.1002/em.22137 (PMC5698699; doi:10.1002/em.22137)

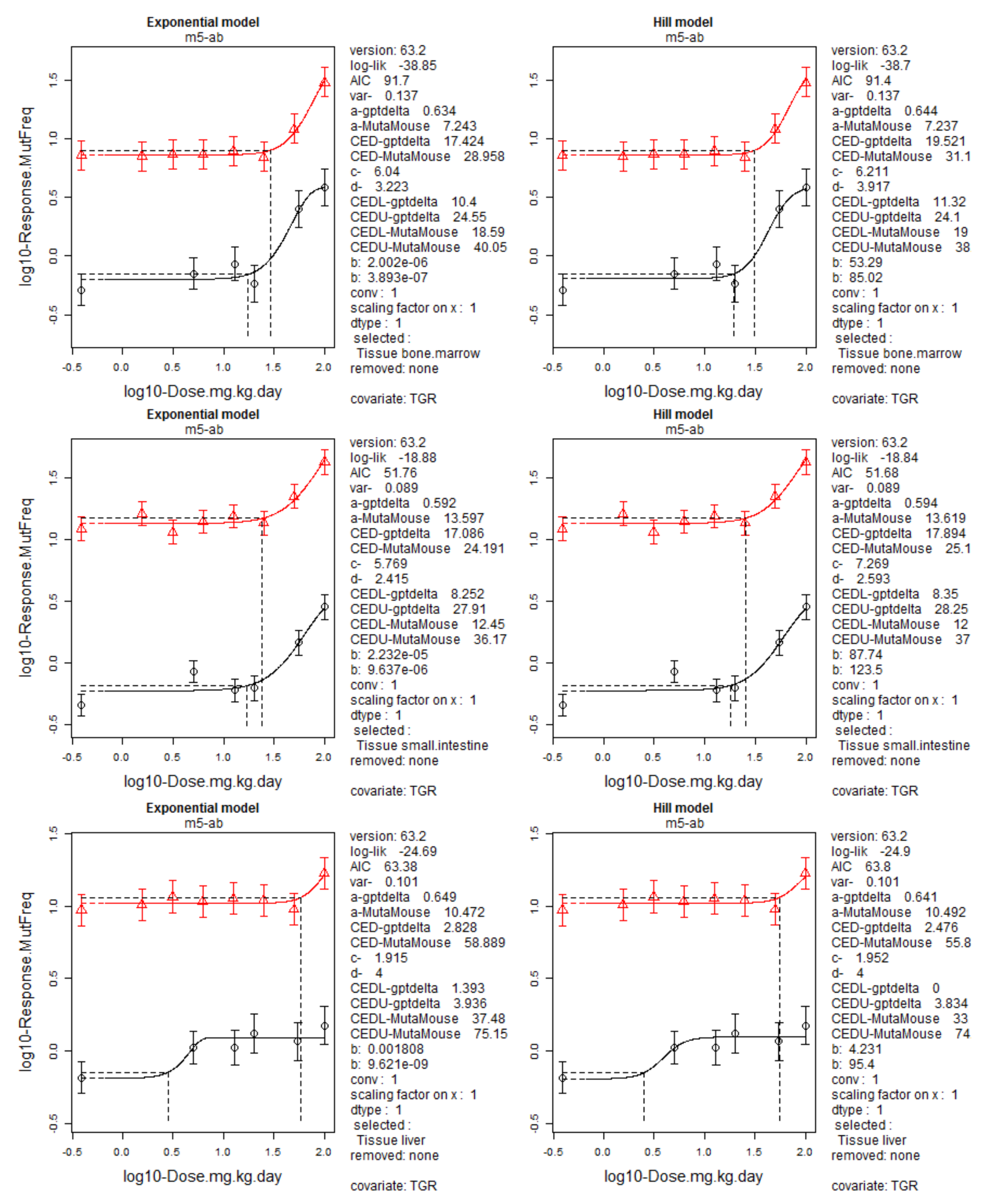

Supplement: Supplementary file 1 — Supporting Information Fig S1 [file EM-58-632-s001.tif]

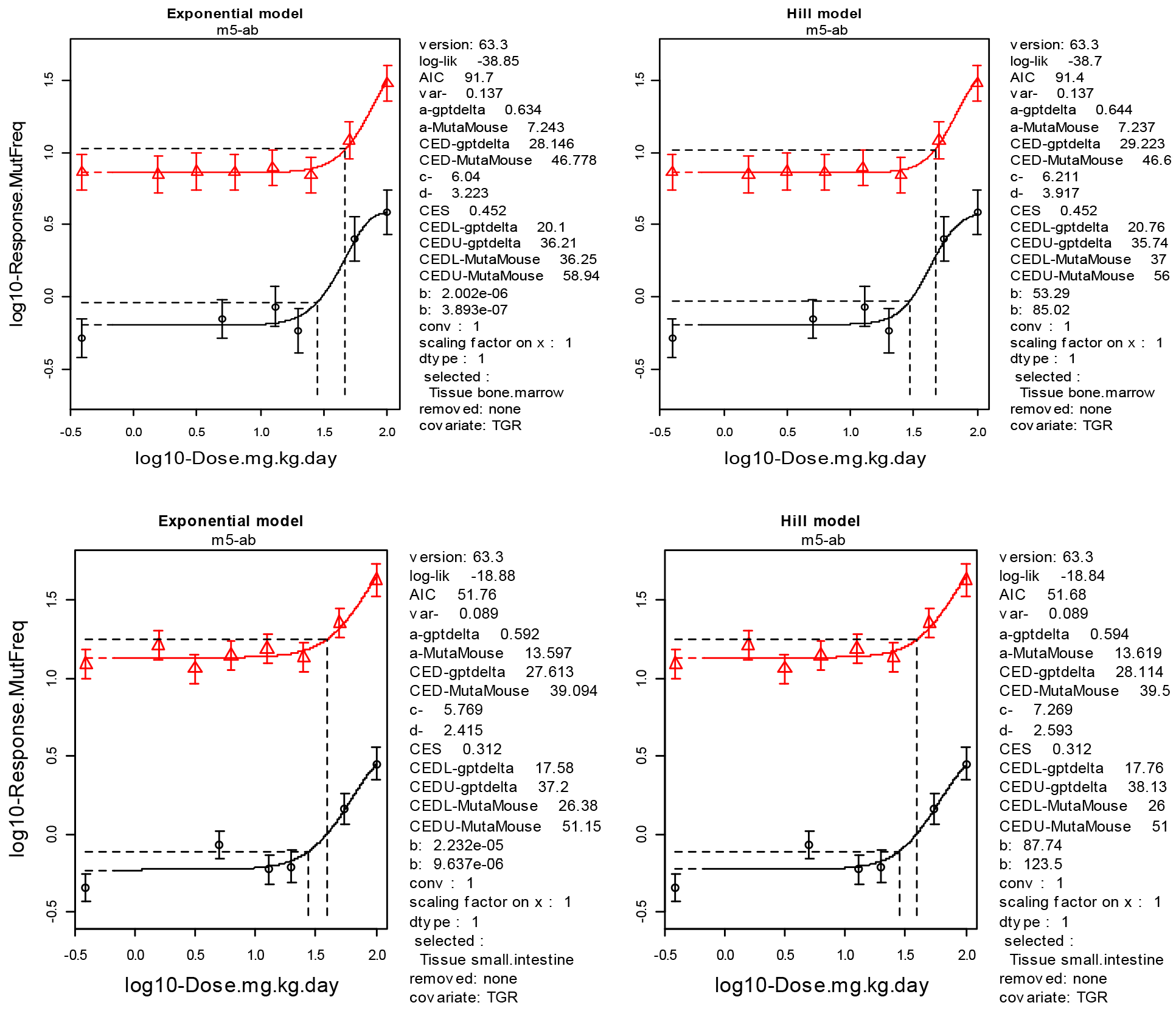

Supplement: Supplementary file 2 — Supporting Information Fig S2 [file EM-58-632-s002.tif]

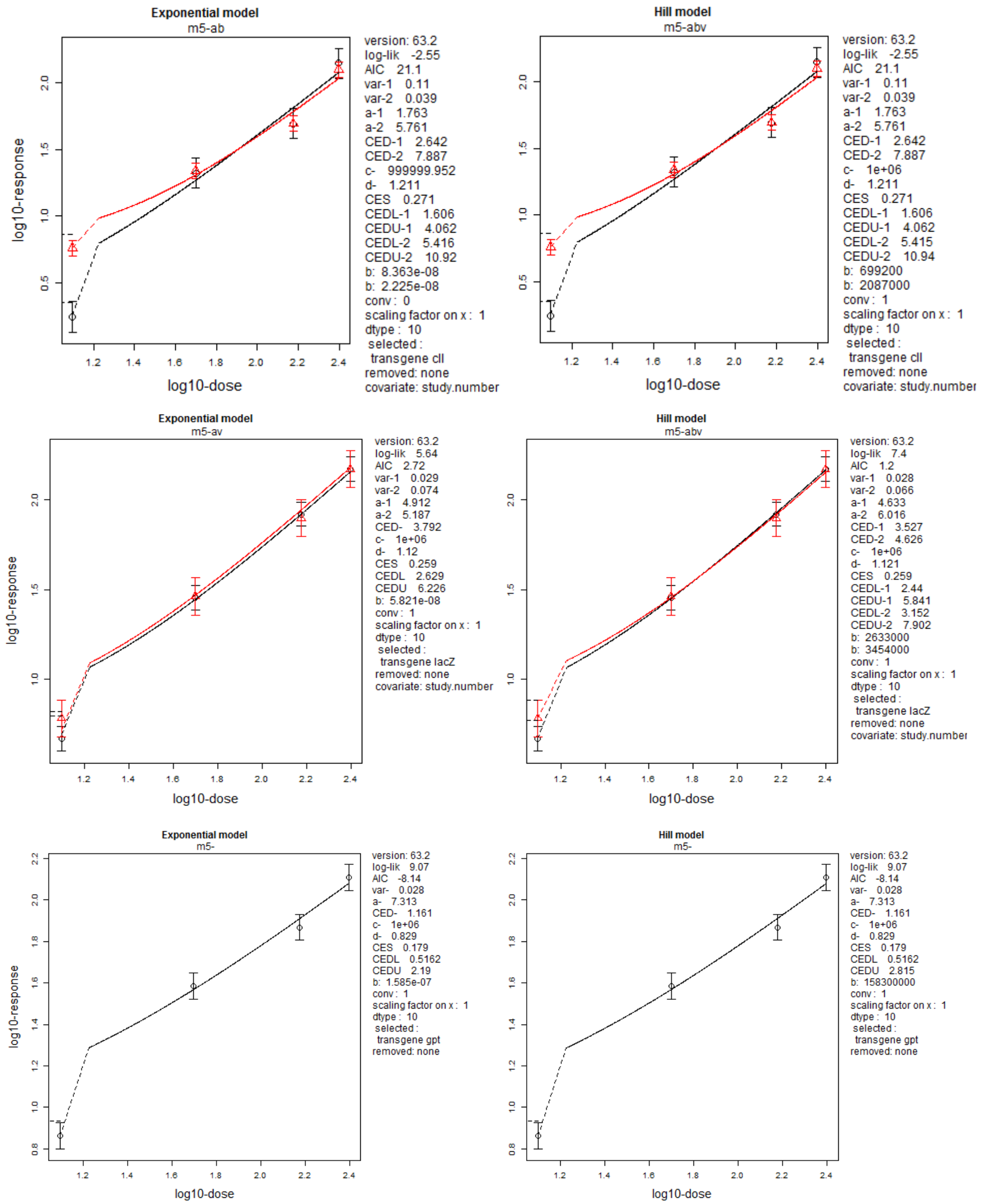

Supplement: Supplementary file 3 — Supporting Information Fig S3 [file EM-58-632-s003.tif]

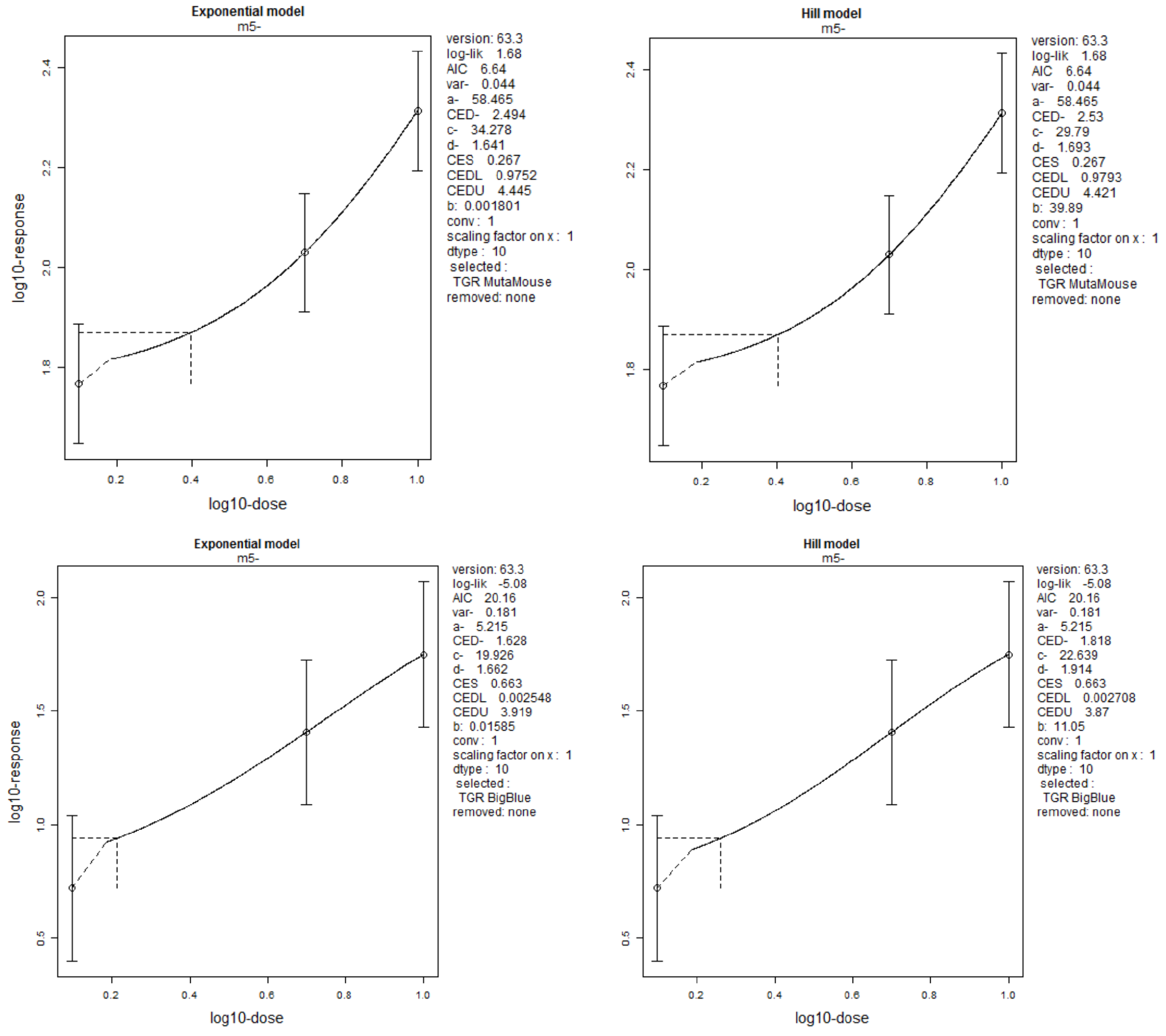

Supplement: Supplementary file 4 — Supporting Information Fig S4 [file EM-58-632-s004.tif]
